# Sucralose Alters Gut Microbiota, Intestinal Metabolites, and Epithelial Serotonergic Responses to Visceral Stimulation in Mice Exposed to Chronic Restraint Stress

**DOI:** 10.3390/nu18162660

**Published:** 2026-08-14

**Authors:** Akira Aoki, Jonathan P. Jacobs, Yoshinori Okamoto

**Affiliations:** 1 Faculty of Pharmacy, Meijo University, 150 Yagotoyama, Tempaku-ku, Nagoya 468-8503, Japan; okmt@meijo-u.ac.jp; 2 Vatche and Tamar Manoukian Division of Digestive Diseases, Department of Medicine, University of California, Los Angeles, CA 90095, USA; 3 Goodman-Luskin Microbiome Center, University of California, Los Angeles, CA 90095, USA; 4 Division of Gastroenterology, Hepatology and Parenteral Nutrition, VA Greater Los Angeles Healthcare System, Los Angeles, CA 90073, USA

**Keywords:** sucralose, chronic restraint stress, host–microbiota interactions, serotonin, tryptophan metabolism, disorders of gut–brain interaction

## Abstract

Background/Objectives: Dietary modifiers of the gut ecosystem may influence intestinal and host responses associated with psychological stress. However, it remains unclear whether sucralose, a widely consumed non-nutritive sweetener, alters host–microbiota interactions following chronic stress exposure. We investigated the effects of short-term experimental sucralose exposure in mice previously subjected to chronic restraint stress (CRS). Methods: Following completion of the CRS protocol, mice received 0.03% or 0.1% sucralose in drinking water for 12 days. Intestinal phenotypes, cecal microbial community structure, microbial metabolites, colonic epithelial gene expression, and epithelial serotonergic responses following allyl isothiocyanate (AITC)-induced visceral stimulation were evaluated. Results: Colon length was significantly reduced in the 0.1% sucralose group, whereas intestinal permeability remained unchanged. Sucralose selectively altered colonic epithelial gene expression and was associated with changes in cecal microbial community structure without marked changes in alpha diversity. The relative abundance of Bacteroides acidifaciens was significantly reduced, particularly in the 0.1% sucralose group. Fecal acetate and cecal tryptophan concentrations were decreased following sucralose exposure. Following AITC-induced visceral stimulation, the CRS + 0.1% sucralose group exhibited reduced immobility behavior together with increased epithelial serotonin and 5-hydroxyindoleacetic acid concentrations. Conclusions: In mice previously exposed to CRS, short-term sucralose exposure was associated with colon shortening, differences in cecal microbial community structure, reduced Bacteroides acidifaciens abundance, decreased fecal acetate and cecal tryptophan concentrations, and altered epithelial serotonergic responsiveness following visceral stimulation. The behavioral and epithelial serotonergic findings suggest that prior stress exposure may influence selected host responses to sucralose. These findings provide a basis for future studies examining how dietary sweeteners modify host–microbiota interactions following chronic stress exposure.

## 1. Introduction

Disorders of gut–brain interaction (DGBIs) comprise a group of conditions characterized by chronic gastrointestinal symptoms arising from complex interactions among neural, immune, microbial, epithelial, and psychosocial factors [1,2]. Psychological stress is considered a major contributor to the development and persistence of DGBIs through its effects on intestinal physiology, barrier function, and the gut microbial ecosystem [2,3]. Irritable bowel syndrome (IBS), one of the most prevalent DGBIs, is characterized by abdominal pain, altered bowel habits, and chronic or recurrent gastrointestinal discomfort [1,2].

The intestinal epithelium serves as a key interface between luminal environmental factors and host physiology [3,4]. Alterations in microbial community structure and microbial metabolites can influence epithelial signaling, serotonin metabolism, and signaling within the gut–brain axis [5,6]. Because psychological stress profoundly affects both intestinal physiology and the gut microbial ecosystem, chronic restraint stress is widely used to model stress-associated intestinal alterations relevant to DGBIs [7,8,9,10]. However, stress alone may not fully explain inter-individual variability in symptom severity, suggesting that dietary and environmental factors may further modify host responses.

Non-nutritive sweeteners are commonly consumed as sugar substitutes, particularly by individuals seeking to reduce caloric intake or modify dietary habits [11,12]. Sucralose is among the most widely used artificial sweeteners and is generally considered safe from a toxicological perspective at currently accepted exposure levels [13]. Nevertheless, experimental studies suggest that sucralose can alter gut microbial composition, microbial metabolism, and host physiological responses [14,15,16]. Dietary modification is a cornerstone of symptom management for many patients with IBS and other DGBIs [17,18]. Despite growing evidence that sucralose influences the gut ecosystem, its effects on intestinal microbial, metabolic, and epithelial responses under stress-associated conditions remain unclear.

In the present study, we investigated whether short-term sucralose exposure administered after completion of the chronic restraint stress (CRS) protocol modifies the intestinal ecosystem and host responses in mice previously exposed to CRS. We hypothesized that short-term sucralose exposure alters microbial, metabolic, and epithelial responses following chronic stress exposure. To test this hypothesis, we used a chronic restraint stress model to evaluate intestinal phenotypes, cecal microbial community structure, microbial metabolites, and epithelial serotonergic responses following visceral stimulation. This study provides new insight into host–microbiota interactions and epithelial serotonergic responses following experimental sucralose exposure in mice previously exposed to chronic stress and expands our understanding of how dietary factors may influence the intestinal ecosystem beyond changes in microbiota composition alone.

## 2. Materials and Methods

### 2.1. Animal Experiments

All animal experiments were conducted in accordance with the Animal Experiment Guidelines of Meijo University and were approved by the Animal Care and Use Committee of Meijo University (approval code: 2026PE36; approval date: 31 March 2026). Male C57BL/6N mice (7 weeks of age) were purchased from Japan SLC Inc. (Shizuoka, Japan) and housed under specific pathogen-free conditions with ad libitum access to food and water. Animals were maintained on a 12 h light/dark cycle under controlled temperature and humidity conditions and were fed a purified AIN-93M diet (Oriental Yeast Co., Ltd., Tokyo, Japan) throughout the study. Following a 1-week acclimation period, mice were randomly assigned to four experimental groups: Non-stressed, chronic restraint stress (CRS) + Vehicle, CRS + 0.03% sucralose (SUC), and CRS + 0.1% SUC (*n* = 6 per group). No formal a priori power analysis was performed. The sample size was selected based on previous experience with this experimental model and preliminary experiments, while considering the ethical principle of minimizing animal use. CRS was performed for 1 h/day on Days 0–4 and 7–11 based on established restraint stress procedures [9]. Mice were placed in restraint tubes constructed from 50 mL polypropylene conical tubes (3.8 cm diameter) with multiple ventilation holes to allow adequate airflow while restricting body movement, similar to previously described restraint apparatuses [10]. Non-stressed mice were handled similarly but were not subjected to restraint. SUC (Tokyo Chemical Industry Co., Ltd., Tokyo, Japan) was dissolved in sterile drinking water at concentrations of 0.03% or 0.1% (*w*/*v*). SUC treatment was initiated immediately after completion of the CRS protocol—that is, after the final restraint session on Day 11—and continued until euthanasia on Day 23. CRS + Vehicle mice received sterile drinking water without SUC. During the treatment period, mice were housed individually to allow accurate measurement of water consumption. Water consumption was monitored throughout the treatment period, and estimated SUC intake was calculated based on body weight and daily water consumption. Body weight was measured throughout the experimental period. Animals were monitored daily for signs of health impairment, and body weight was monitored throughout the experimental period. Humane endpoints were predefined in the approved animal protocol; animals showing marked deterioration in general condition or abnormal changes in body weight were to be humanely euthanized. No animals met the humane endpoint criteria during the study. At the end of the study, intestinal permeability was evaluated using a fluorescein isothiocyanate-dextran (FITC-dextran) assay. Mice were subsequently euthanized by overdose of inhaled isoflurane administered in a closed chamber. The delivered isoflurane concentration was not quantified. Loss of spontaneous respiration was confirmed before terminal blood collection, and death was confirmed by the continued absence of spontaneous respiration prior to tissue collection. Blood, fecal samples, cecal contents, and colonic tissues were subsequently collected. Colon length was measured at necropsy. Colonic epithelial scrapings were obtained by gentle mucosal scraping after removal of luminal contents with phosphate-buffered saline (PBS) and were used for gene expression analysis. Cecal contents were collected for microbiota and metabolite analyses, and fecal samples were collected for SCFA measurements.

For the allyl isothiocyanate (AITC) challenge experiment, an independent cohort of mice was prepared using the same CRS and SUC treatment protocol. The primary AITC cohort consisted of Non-stressed, CRS + Vehicle, and CRS + 0.1% SUC groups (*n* = 6 per group). On Day 23, mice received intracolonic administration of AITC, and behavioral responses were recorded for 15 min. Colonic epithelial scrapings were collected immediately after behavioral assessment for measurement of serotonin (5-HT) and 5-hydroxyindoleacetic acid (5-HIAA). To evaluate the effects of sucralose in the absence of stress, an additional Non-stressed + 0.1% SUC group (*n* = 6) was examined using the same AITC challenge protocol.

### 2.2. Intestinal Permeability Assay

Intestinal permeability was assessed using FITC-dextran (4 kDa; Sigma-Aldrich, St. Louis, MO, USA) as previously described with minor modifications [19]. Mice were fasted for 9 h with free access to water before administration. FITC-dextran was dissolved in PBS at 50 mg/mL and administered by oral gavage at a dose of 440 mg/kg body weight. Four hours after FITC-dextran administration, mice were euthanized as described above, and terminal blood was collected from the vena cava. Blood samples were centrifuged at 2000× *g* for 10 min at 4 °C to obtain plasma. Plasma samples were diluted fourfold with water, and fluorescence was measured in duplicate using a black 96-well plate on an EnSight multimode plate reader (PerkinElmer, Waltham, MA, USA) with excitation and emission wavelengths of approximately 485 and 530 nm, respectively. Plasma FITC-dextran concentrations were calculated using a four-parameter logistic standard curve generated from serial dilutions of FITC-dextran. Standards were prepared in the same matrix ratio as the samples. Blank-corrected fluorescence values were used for quantification, and calculated concentrations were corrected for the fourfold dilution to obtain plasma FITC-dextran concentrations.

### 2.3. Quantitative Real-Time RT-PCR Analysis

Total RNA was extracted from colonic epithelial scrapings using a spin-column kit (ISOSPIN Cell & Tissue RNA; Nippon Gene Co., Tokyo, Japan) according to the manufacturer’s instructions. First-strand cDNA was synthesized from 500 ng of total RNA using ReverTra Ace qPCR RT Master Mix (Toyobo Co., Ltd., Osaka, Japan). Quantitative real-time PCR was performed using THUNDERBIRD NEXT SYBR qPCR Mix (Toyobo Co., Ltd.) on a LightCycler 480 System II (Roche Diagnostics GmbH, Mannheim, Germany). Primer sequences were selected from previously published studies when available [20,21,22] or designed based on reference sequences obtained from OriGene Technologies (Rockville, MD, USA). All primer sequences are listed in Supplementary Table S1. Amplification was performed with an initial denaturation step at 95 °C for 30 s, followed by 40 cycles of 95 °C for 5 s and 60 °C for 10 s. All reactions were performed in duplicate, and the mean Ct value of the duplicate measurements was used for subsequent analyses. Relative expression values for graphical presentation were calculated using the comparative Ct (2^−ΔΔCt^) method, with *Gapdh* used as the reference gene and the mean ΔCt value of the CRS + Vehicle group used as the calibrator [23]. Statistical comparisons were performed using ΔCt values, as described below. Primer specificity was confirmed by melting curve analysis to verify the presence of a single amplification product.

### 2.4. 16S rRNA Gene Sequencing and Microbiota Analysis

Genomic DNA was extracted from cecal contents using the NucleoSpin DNA Stool Kit (Macherey-Nagel, Düren, Germany) according to the manufacturer’s instructions. The extracted DNA samples were submitted to Novogene Co., Ltd. (Beijing, China) for full-length 16S rRNA gene sequencing. The V1–V9 region of the bacterial 16S rRNA gene was amplified using universal primers 27F (5′-AGRGTTYGATYMTGGCTCAG-3′) and 1492R (5′-GYTACCTTGTTACGACTT-3′). Libraries were prepared and sequenced on the PacBio Sequel II platform using circular consensus sequencing (CCS) technology. After quality filtering, a total of 346,862 high-quality CCS reads were obtained, corresponding to 10,333–19,819 clean reads per sample, with an average read length of approximately 1458 bp. Amplicon sequence variants (ASVs) were inferred from CCS reads using the DADA2 package (version 1.36.0) in R (version 4.5.1), including quality filtering, denoising, and chimera removal [24,25]. Taxonomic assignment was primarily performed using a custom GTDB 50K reference database constructed from GTDB release R226 [26]. SILVA release 138.2 was additionally used to support taxonomic annotation and nomenclature [27]. Microbial community analyses were conducted using the phyloseq package (version 1.52.0) [28]. Alpha diversity was evaluated using the Chao1 richness estimator and Shannon diversity index. Beta diversity was assessed using Bray–Curtis dissimilarity and visualized by non-metric multidimensional scaling (NMDS). Differences in microbial community composition among groups were evaluated by PERMANOVA using the adonis2 function in the vegan package with 999 permutations [29]. Pairwise PERMANOVA was performed using the pairwise.adonis function in the pairwiseAdonis package (version 0.4.1), and *p* values were adjusted using the Benjamini–Hochberg method. Multivariable association analysis was performed using the MaAsLin2 package (version 1.22.0) in R [30]. To identify bacterial ASVs associated with SUC exposure, the analysis was restricted to CRS-exposed mice. Treatment was included as the sole fixed effect, with CRS + Vehicle specified as the reference group. Features with a minimum abundance of 0.01 and a minimum prevalence of 20% were retained. The *p* values were adjusted using the Benjamini–Hochberg false discovery rate (FDR) procedure. For visualization, ASVs assigned to named bacterial species that met an exploratory FDR threshold (*q* ≤ 0.25) in at least one sucralose comparison were included in the forest plot. For species-level analysis of *Bacteroides acidifaciens*, the relative abundances of all ASVs assigned to *B. acidifaciens* were summed for each sample. Before statistical analysis, a pseudocount corresponding to one-half of the minimum nonzero relative abundance was added, and the resulting values were log10-transformed. Group differences were evaluated using one-way ANOVA followed by Dunnett’s multiple-comparison test, with CRS + Vehicle as the reference group.

### 2.5. LC–MS/MS Measurement of Fecal SCFAs

Fecal samples were collected at necropsy and stored at −80 °C until analysis. Six SCFAs, including acetic acid (AA), propionic acid (PA), butyric acid (BA), isobutyric acid (IBA), valeric acid (VA), and isovaleric acid (IVA), were quantified as previously described with minor modifications [31,32,33]. Fecal samples were weighed immediately before extraction. An internal standard mixture containing stable isotope-labeled SCFAs (SBR00034; Sigma-Aldrich) was diluted 1000-fold in ice-cold 80% methanol. For metabolite extraction, 400 µL of the internal standard-containing extraction solvent was added to a 2 mL screw-cap tube containing the sample and 100–150 mg of 1 mm glass beads. Samples were homogenized using a bead-beating procedure consisting of three cycles of 1 min vortexing at maximum speed, separated by 30 s cooling intervals on ice. Following homogenization, samples were centrifuged at 15,000× *g* for 10 min at 4 °C. The supernatants were collected and filtered through 0.22 µm PTFE syringe filters. SCFAs were derivatized using the 3-nitrophenylhydrazine (3-NPH) method. Briefly, 60 µL of filtered sample or calibration standard was mixed with 60 µL of 200 mM 3-NPH·HCl prepared in 50% methanol. Subsequently, 24 µL of freshly prepared 120 mM 1-ethyl-3-(3-dimethylaminopropyl) carbodiimide solution in 6% pyridine/methanol was added and mixed immediately. After incubation at 40 °C with shaking at 800 rpm for 30 min, samples were centrifuged at 15,000× *g* for 10 min, and the resulting supernatants were subjected to LC–MS/MS analysis. LC–MS/MS analysis was performed using a UHPLC system coupled to a triple quadrupole mass spectrometer (QTRAP 6500+; Sciex, Framingham, MA, USA) equipped with an electrospray ionization source operating in negative-ion mode. Chromatographic separation was achieved using an ACQUITY UPLC CSH C18 column (100 × 2.1 mm, 1.7 µm; Waters, Milford, MA, USA) maintained at 40 °C. The flow rate was 0.5 mL/min, and the injection volume was 2 µL. Mobile phase A consisted of water containing 0.1% formic acid, and mobile phase B consisted of acetonitrile. The gradient program was as follows: 10% B from 0–5.0 min, increased linearly to 40% B at 10.5 min, increased to 98% B at 10.51 min and held until 12.5 min, then returned to 10% B at 12.51 min and equilibrated until 14.5 min. Detection was performed in multiple reaction monitoring (MRM) mode. Optimized MRM transitions, declustering potentials, collision energies, and retention times are listed in Supplementary Table S2. SCFA concentrations were calculated from calibration curves using analyte-to-internal-standard peak-area ratios and normalized to fecal wet weight.

### 2.6. LC–MS/MS Measurement of Cecal Tryptophan-Related Metabolites

Cecal contents were collected at necropsy and stored at −80 °C until analysis. Tryptophan-related metabolites, including tryptophan (Trp), kynurenine (Kyn), kynurenic acid (KA), indole-3-acetic acid (IAA), indole-3-lactic acid (ILA), and indole-3-propionic acid (IPA), were quantified by LC–MS/MS using stable isotope-labeled internal standards as previously described with modifications [34,35]. Cecal samples were weighed and transferred to a 2 mL screw-cap tube containing 1 mm glass beads. Samples were homogenized in 400 µL of ice-cold 80% methanol containing isotope-labeled internal standards (Trp-d5, Kyn-d4, and IAA-d5) using a bead-beating procedure consisting of three cycles of 1 min vortexing at maximum speed separated by 30 s cooling intervals on ice. After incubation on ice for 10–15 min, samples were centrifuged at 15,000× *g* for 10 min at 4 °C. The supernatants were transferred to new tubes, centrifuged again at 15,000× *g* for 5 min at 4 °C, and filtered through 0.22 µm PTFE membrane filters prior to LC–MS/MS analysis. LC–MS/MS analysis was performed using a UHPLC system coupled to a triple quadrupole mass spectrometer (QTRAP 6500+; Sciex) equipped with an electrospray ionization source operating in positive-ion mode. Chromatographic separation was achieved using a Kinetex EVO C18 column (100 × 2.1 mm, 2.6 µm; Phenomenex, Torrance, CA, USA) maintained at 40 °C. The flow rate was 0.6 mL/min, and the injection volume was 2 µL. Mobile phase A consisted of water containing 0.1% formic acid, and mobile phase B consisted of methanol. The gradient program was as follows: 3% B from 0 to 2 min; increased linearly to 60% B from 2 to 10 min; increased to 98% B at 10.01 min and held until 12 min; returned to 3% B at 12.01 min and equilibrated until 14.5 min. Detection was performed in MRM mode. Optimized MRM transitions and compound-specific parameters are listed in Supplementary Table S3. Metabolite concentrations were calculated from calibration curves using analyte-to-internal-standard peak-area ratios and normalized to cecal content wet weight.

### 2.7. AITC-Induced Visceral Stimulation Test

Behavioral responses to visceral stimulation were evaluated using intracolonic administration of AITC (Tokyo Chemical Industry). Mice were individually placed in transparent acrylic observation chambers (20 × 20 cm) and allowed to acclimate for 15 min prior to testing. AITC was dissolved in corn oil and administered intracolonically at a concentration of 2% (*v*/*v*) in a volume of 50 μL using a flexible catheter with a silicone-covered tip to minimize mucosal injury. The catheter was inserted approximately 3 cm proximal to the anal verge, based on previously described intracolonic AITC-induced visceral nociception protocols in mice [36]. This condition was selected based on preliminary experiments to avoid excessive behavioral responses in non-stressed mice. Immediately following AITC administration, mice were returned to the observation chambers and monitored for 15 min. All behavioral sessions were video-recorded. Behavioral responses were subsequently evaluated independently by two observers blinded to the experimental groups. Three behavioral endpoints were quantified: total immobility time, licking behavior directed toward the abdomen or perianal region, and crossing activity. Immobility was defined as the cumulative duration during which the mouse remained stationary except for respiratory movements. Licking behavior was quantified as the total number of licking episodes directed toward the abdomen or perianal region. For assessment of locomotor activity, a floor marked with two perpendicular center lines was placed in the observation chamber, and crossing activity was quantified as the number of times the animal crossed either center line. These parameters were used as complementary indices of the behavioral response to visceral stimulation. Immediately after completion of the behavioral assessment, mice were euthanized by cervical dislocation to enable rapid collection of colonic epithelial tissue for measurement of 5-HT and 5-HIAA.

### 2.8. HPLC–ECD Measurement of Colonic Epithelial 5-HT and 5-HIAA

Colonic epithelial concentrations of 5-HT and its primary metabolite 5-HIAA were quantified by high-performance liquid chromatography with electrochemical detection (HPLC–ECD) as previously described with modifications [37]. 5-HT hydrochloride (Abcam, Cambridge, UK) and 5-HIAA (Tokyo Chemical Industry Co.) were used as calibration standards. Isoproterenol hydrochloride (ISO; Tokyo Chemical Industry Co., Ltd.) was used as an internal standard. Colonic epithelial scrapings were collected immediately after the 15 min behavioral observation period following AITC administration, rapidly frozen in liquid nitrogen, and stored at −80 °C until analysis. For metabolite extraction, samples were homogenized in ice-cold 0.1 M perchloric acid containing ISO (100 nM) as an internal standard. Briefly, epithelial samples were transferred to microcentrifuge tubes containing glass beads and 400 μL of extraction solution. Samples were vortexed repeatedly on ice and incubated for 10 min before centrifugation at 16,000× *g* for 10 min at 4 °C. The supernatants were collected and centrifuged again under the same conditions. The resulting supernatants were diluted 10-fold with 0.1 M perchloric acid containing ISO (100 nM) prior to analysis. Chromatographic separation was performed using a CA-5ODS column (2.1 × 150 mm; Eicom, Kyoto, Japan) maintained at 25 °C. The mobile phase consisted of 100 mM citrate buffer (pH 3.5) and methanol (85:15, *v*/*v*) containing sodium 1-octanesulfonate (500 mg/L) and EDTA-2Na (50 mg/L). The flow rate was 230 μL/min. Electrochemical detection was performed using an ECD-800 detector (Eicom) with the working electrode potential set at +450 mV. The HPLC system consisted of an LC-20AB pump (Shimadzu, Kyoto, Japan). Analyte concentrations were quantified using calibration curves based on analyte-to-internal-standard peak-area ratios and normalized to the wet weight of epithelial samples. Peak identities were confirmed by comparison of retention times with those of corresponding standards.

### 2.9. Statistical Analysis

Data are presented as the mean ± SEM unless otherwise indicated. Statistical analyses were performed using R (version 4.5.1). For comparisons among multiple groups, one-way ANOVA followed by Dunnett’s multiple-comparison test was used, with CRS + Vehicle as the reference group. Alpha-diversity metrics were analyzed using the Kruskal–Wallis test followed by Dunn’s multiple-comparison test with Benjamini–Hochberg correction. Microbial beta-diversity analyses, including PERMANOVA and pairwise PERMANOVA, were performed as described above. Multivariable association analysis was performed using MaAsLin2 as described above. For microbiome analyses involving feature-wise or pairwise multiple testing, *p* values were adjusted using the Benjamini–Hochberg FDR procedure and are reported as q values. Unless otherwise stated, *p* < 0.05 or q < 0.05, as applicable, was considered statistically significant. A threshold of q ≤ 0.25 was used only for exploratory visualization of MaAsLin2 associations. Statistical analyses of gene-expression data were performed using ΔCt values (Ct_target − Ct_Gapdh). Comparisons between two groups in the non-stressed AITC cohort were performed using Welch’s two-tailed unpaired *t*-test. In addition to hypothesis-testing results, descriptive statistics, effect estimates with 95% confidence intervals, and standardized effect sizes (Cohen’s d) for the principal study outcomes are provided in Supplementary Table S4. For this table, Cohen’s d was calculated on the scale used for statistical analysis; ΔCt values were used for gene-expression outcomes, and log10-transformed relative abundance values were used for Bacteroides acidifaciens.

## 3. Results

### 3.1. Sucralose Exposure Is Associated with Colon Shortening Without Altering Intestinal Permeability in CRS-Exposed Mice

**Figure 1:**
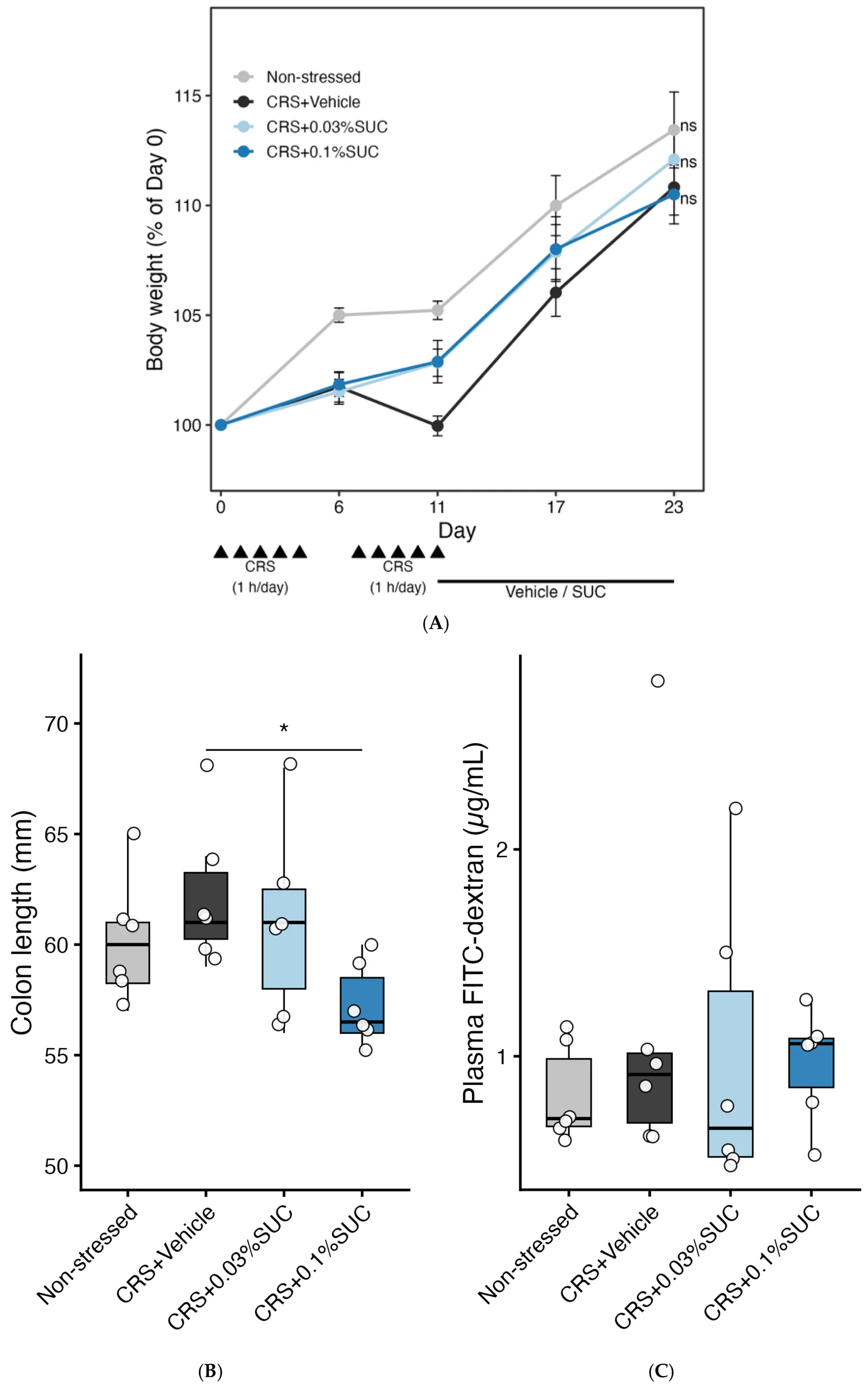
Sucralose exposure induces colon shortening without affecting body weight gain or intestinal permeability in CRS-exposed mice. Mice were subjected to chronic restraint stress (CRS; 1 h/day) on Days 0–4 and 7–11. Black triangles indicate CRS sessions. Immediately after the final restraint session on Day 11, mice received vehicle, 0.03% sucralose (SUC), or 0.1% SUC in drinking water until euthanasia on Day 23. Non-stressed mice received sterile drinking water without sucralose throughout the experimental period. (**A**) Body weight changes during the experimental period, expressed as a percentage of body weight on Day 0. Data are presented as mean ± SEM. (**B**) Colon length measured at necropsy on Day 23. (**C**) Intestinal permeability assessed by the fluorescein isothiocyanate-dextran (FITC-dextran) assay at the end of the experiment. Circles represent individual mice, and box plots indicate the median and interquartile range. Statistical comparisons were performed using one-way ANOVA followed by Dunnett’s multiple-comparison test with CRS + Vehicle as the reference group. For body weight, statistical comparisons were performed using relative body-weight values on Day 23. Significance is indicated by *p* < 0.05 (*); ns, not significant.

**Figure 2:**
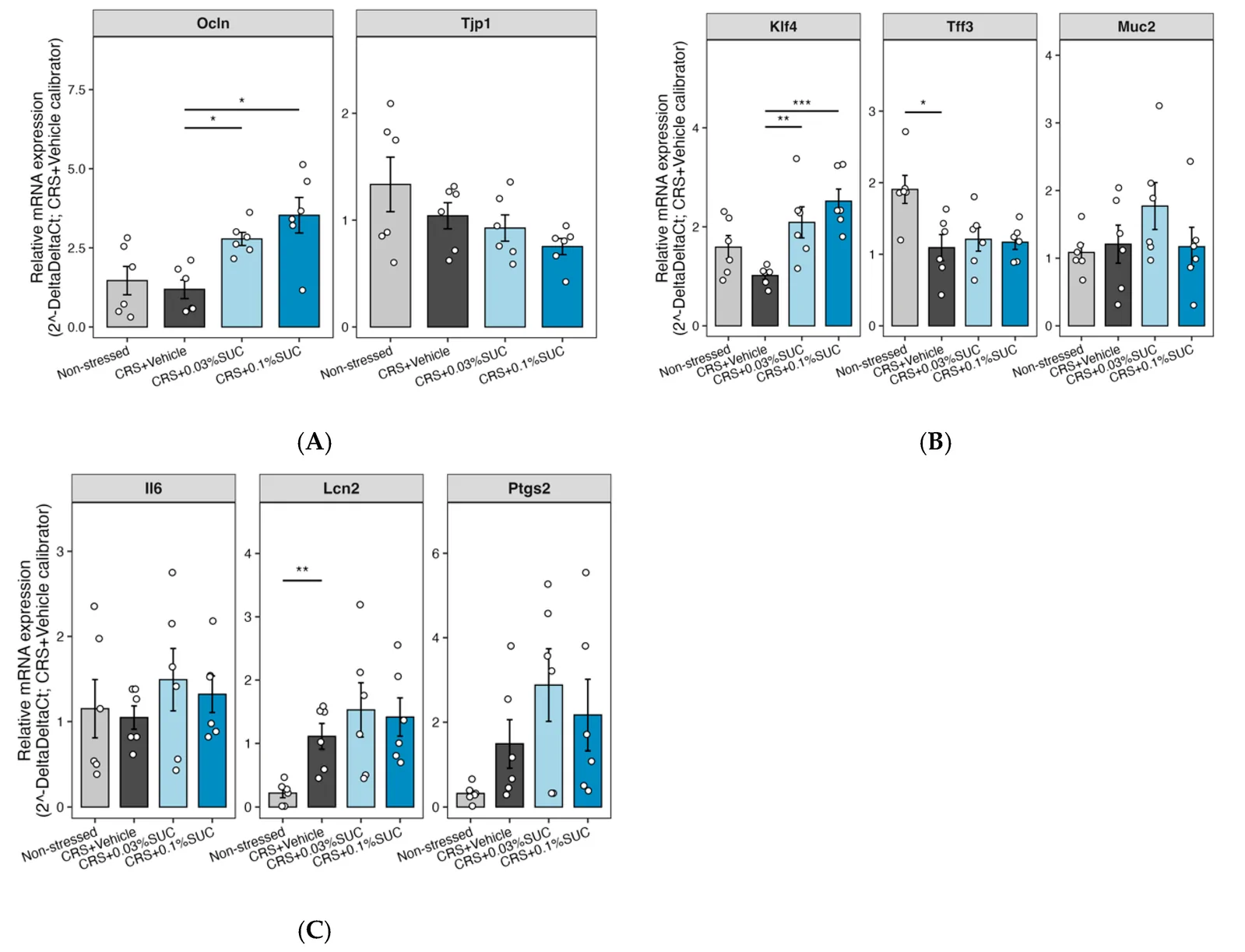
Sucralose alters colonic epithelial expression of genes associated with barrier function, mucosal defense, and epithelial stress responses in CRS-exposed mice. (**A**) Relative mRNA expression of colonic epithelial genes associated with barrier function (*Ocln* and *Tjp1*); (**B**) relative mRNA expression of genes associated with goblet cell differentiation and mucosal defense (*Klf4*, *Tff3*, and *Muc2*); (**C**) relative mRNA expression of genes associated with inflammatory and epithelial stress responses (*Il6*, *Lcn2*, and *Ptgs2*). Mice were subjected to chronic restraint stress (CRS; 1 h/day) on Days 0–4 and 7–11. Immediately after the final restraint session on Day 11, mice received vehicle, 0.03% sucralose (SUC), or 0.1% SUC in drinking water until euthanasia on Day 23. Non-stressed mice received sterile drinking water without sucralose throughout the experimental period. Relative expression was calculated using the 2^−ΔΔCt^ method. Bars represent mean ± SEM, and circles represent individual mice. Statistical comparisons were performed on ΔCt values using one-way ANOVA followed by Dunnett’s multiple-comparison test, with CRS + Vehicle as the reference group. Significance is indicated by *p* < 0.05 (*), *p* < 0.01 (**), and *p* < 0.001 (***).

Following the final CRS session, mice received vehicle, 0.03% SUC, or 0.1% SUC in drinking water for 12 days (Figure 1A). No significant differences in water intake were observed among the CRS-exposed groups during the treatment period. Based on individual water consumption, the estimated sucralose exposure was 39.4 ± 2.1 mg/kg/day in the 0.03% SUC group and 136.8 ± 8.4 mg/kg/day in the 0.1% SUC group. Body weight trajectories were similar among the experimental groups, and relative body weight on Day 23 did not differ significantly (Figure 1A). Colon length was significantly reduced in the CRS + 0.1% SUC group compared with the CRS + Vehicle group, whereas no significant differences were observed in the Non-stressed or CRS + 0.03% SUC groups (Figure 1B). In contrast, intestinal permeability assessed by the FITC-dextran assay was not significantly altered by CRS or sucralose treatment (Figure 1C). We next examined the expression of epithelial genes associated with barrier function and mucosal defense. Tff3 expression was significantly reduced by CRS exposure, whereas Ocln and Klf4 expression were significantly increased in both the CRS + 0.03% SUC and CRS + 0.1% SUC groups. No significant differences were observed for Tjp1 or Muc2 (Figure 2A,B). To examine whether colon shortening was accompanied by changes in epithelial inflammatory responses, expression of Il6, Lcn2, and Ptgs2 was quantified. CRS exposure significantly increased epithelial Lcn2 expression, whereas Il6 expression remained unchanged. Ptgs2 expression also tended to increase in CRS-exposed mice, although the difference did not reach statistical significance (Dunnett-adjusted *p* = 0.093). Neither 0.03% nor 0.1% SUC further altered the expression of these genes (Figure 2C).

### 3.2. Sucralose Alters Gut Microbial Community Structure and Reduces the Relative Abundance of Bacteroides Acidifaciens in CRS-Exposed Mice

**Figure 3:**
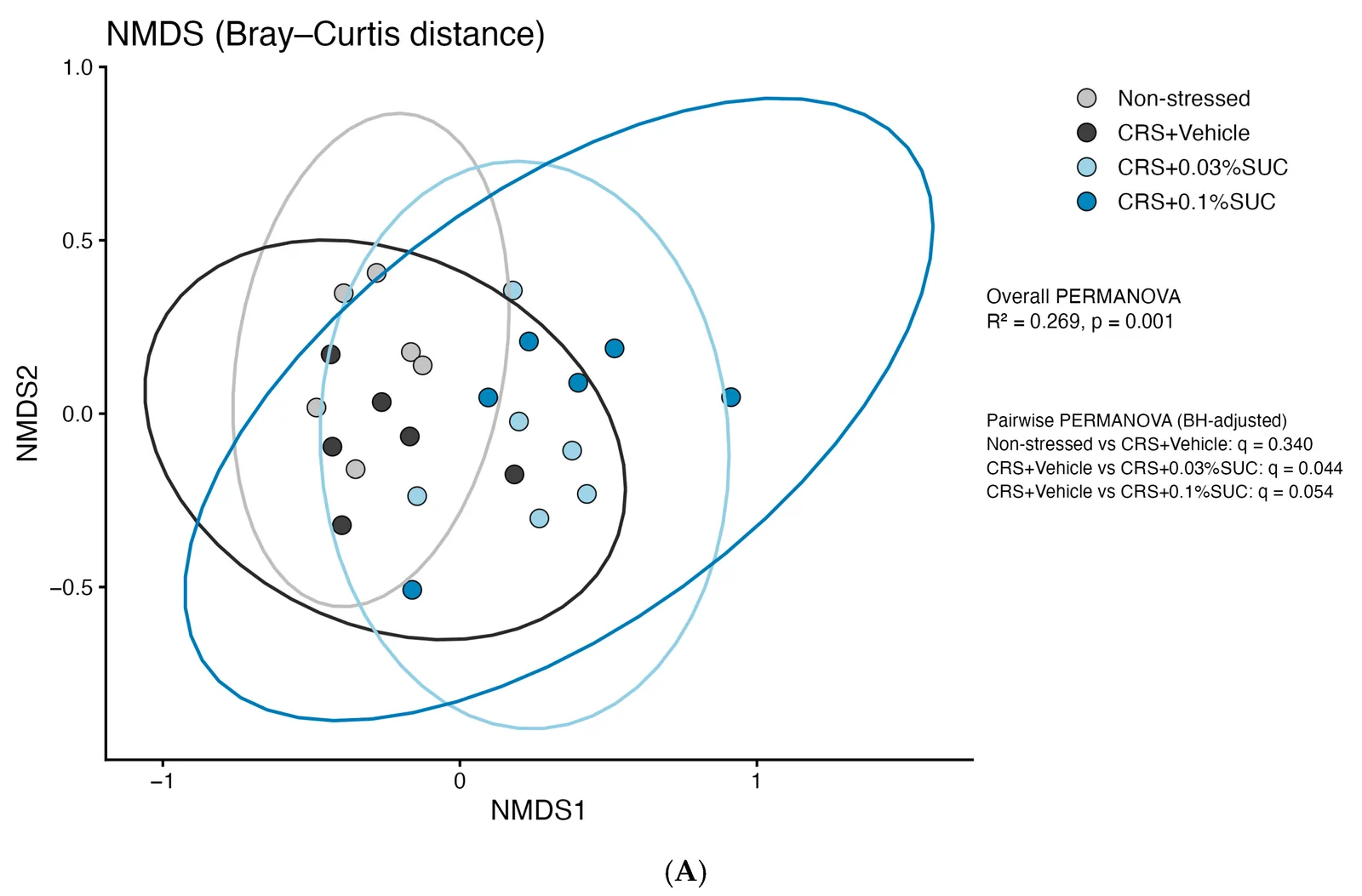

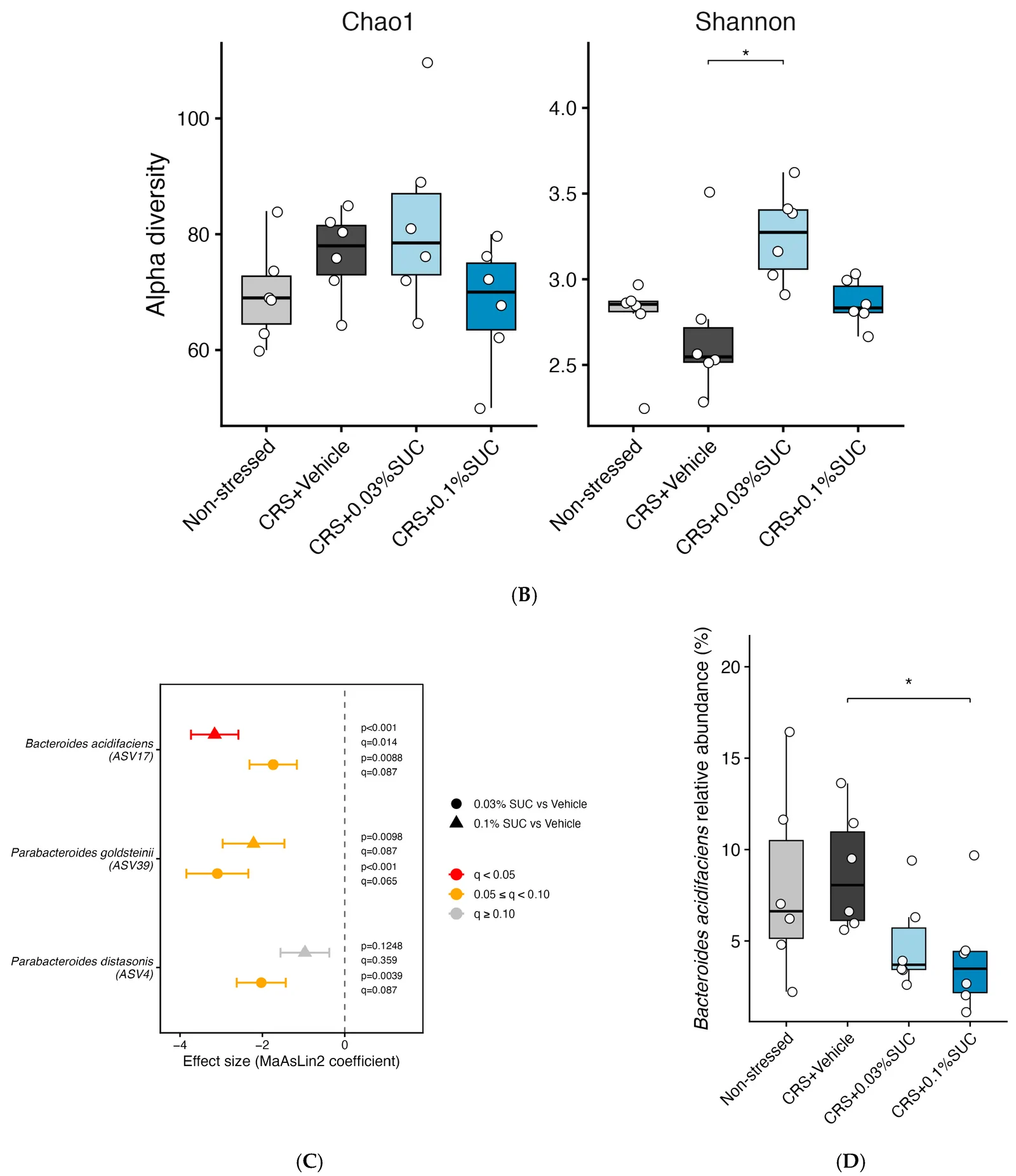
Sucralose is associated with changes in cecal microbial community structure and taxonomic composition in CRS-exposed mice. (**A**) Nonmetric multidimensional scaling (NMDS) based on Bray–Curtis dissimilarity showing differences in cecal microbial community composition among the Non-stressed, chronic restraint stress (CRS) + Vehicle, CRS + 0.03% sucralose (SUC), and CRS + 0.1% SUC groups. Each circle represents an individual mouse, and colored ellipses indicate the distribution of each experimental group. Overall group differences were assessed using permutational multivariate analysis of variance (PERMANOVA). Selected pairwise comparisons were evaluated using pairwise PERMANOVA with Benjamini–Hochberg correction, and the resulting *q* values are shown in the figure. (**B**) Alpha diversity of the cecal microbiota assessed using the Shannon diversity index and Chao1 richness estimator. Box plots indicate the median and interquartile range, and circles represent individual mice. Overall group differences were assessed using the Kruskal–Wallis test. Pairwise comparisons versus CRS + Vehicle were performed using Dunn’s multiple-comparison test with Benjamini–Hochberg correction. (**C**) Forest plot of taxonomically identified bacterial ASVs associated with SUC exposure in MaAsLin2 analyses. Analyses were restricted to CRS-exposed mice, and separate comparisons were performed for the 0.03% SUC and 0.1% SUC groups, with CRS + Vehicle specified as the reference group. ASVs meeting the exploratory threshold of *q* ≤ 0.25 in at least one comparison are shown. Circles and triangles represent the 0.03% SUC versus Vehicle and 0.1% SUC versus Vehicle comparisons, respectively. Colors indicate FDR categories (*q* < 0.05, 0.05 ≤ *q* < 0.10, and *q* ≥ 0.10). Point estimates represent MaAsLin2 coefficients, horizontal lines indicate ± standard error, and the vertical dashed line indicates a coefficient of zero. Numerical labels indicate nominal *p* values and false discovery rate-adjusted *q* values. (**D**) Relative abundance of *Bacteroides acidifaciens*, calculated by summing the relative abundances of all amplicon sequence variants (ASVs) assigned to *B. acidifaciens*. Statistical comparisons were performed on log10-transformed relative abundance values using one-way ANOVA followed by Dunnett’s multiple-comparison test with CRS + Vehicle as the reference group. Circles represent individual mice. Significance is indicated by *p* < 0.05 (*).

To determine whether SUC exposure altered the gut microbiota under CRS conditions, we analyzed cecal microbial community composition by full-length 16S rRNA gene sequencing. NMDS based on Bray–Curtis dissimilarity demonstrated significant differences in overall microbial community composition among the experimental groups (PERMANOVA, R^2^ = 0.269, *p* = 0.001; Figure 3A). Pairwise PERMANOVA showed no significant difference between Non-stressed and CRS + Vehicle mice (q = 0.340). Compared with CRS + Vehicle, microbial community composition differed significantly in the CRS + 0.03% SUC group (q = 0.044), whereas the difference did not reach statistical significance in the CRS + 0.1% SUC group (q = 0.054). Alpha diversity was largely unchanged, although the Shannon diversity index was increased in the CRS + 0.03% SUC group compared with the CRS + Vehicle group; no significant differences were observed in Chao1 richness (Figure 3B).

To identify individual ASVs associated with SUC exposure, MaAsLin2 analysis was performed in CRS-exposed mice only, with treatment included as a fixed effect and CRS + Vehicle used as the reference group. For visualization, the forest plot includes ASVs assigned to named bacterial species that met an exploratory FDR threshold of q ≤ 0.25 in at least one SUC comparison (Figure 3C). Among these taxa, Bacteroides acidifaciens ASV17 was the only ASV that met the conventional FDR threshold of q < 0.05, showing a marked negative association with 0.1% SUC treatment (coefficient = −3.160, *p* = 6.45 × 10^−5^, q = 0.014). The same ASV was also negatively associated with 0.03% SUC treatment at the exploratory threshold (coefficient = −1.739, *p* = 0.0088, q = 0.087). We therefore quantified the species-level abundance of *B. acidifaciens* by summing the relative abundances of all ASVs assigned to this species. Species-level *B. acidifaciens* abundance was significantly reduced in the CRS + 0.1% SUC group compared with the CRS + Vehicle group (Dunnett-adjusted *p* = 0.034), whereas the reduction in the CRS + 0.03% SUC group did not reach statistical significance (Dunnett-adjusted *p* = 0.174; Figure 3D).

### 3.3. Sucralose Exposure Alters Fecal SCFA and Cecal Tryptophan Profiles

**Figure 4:**
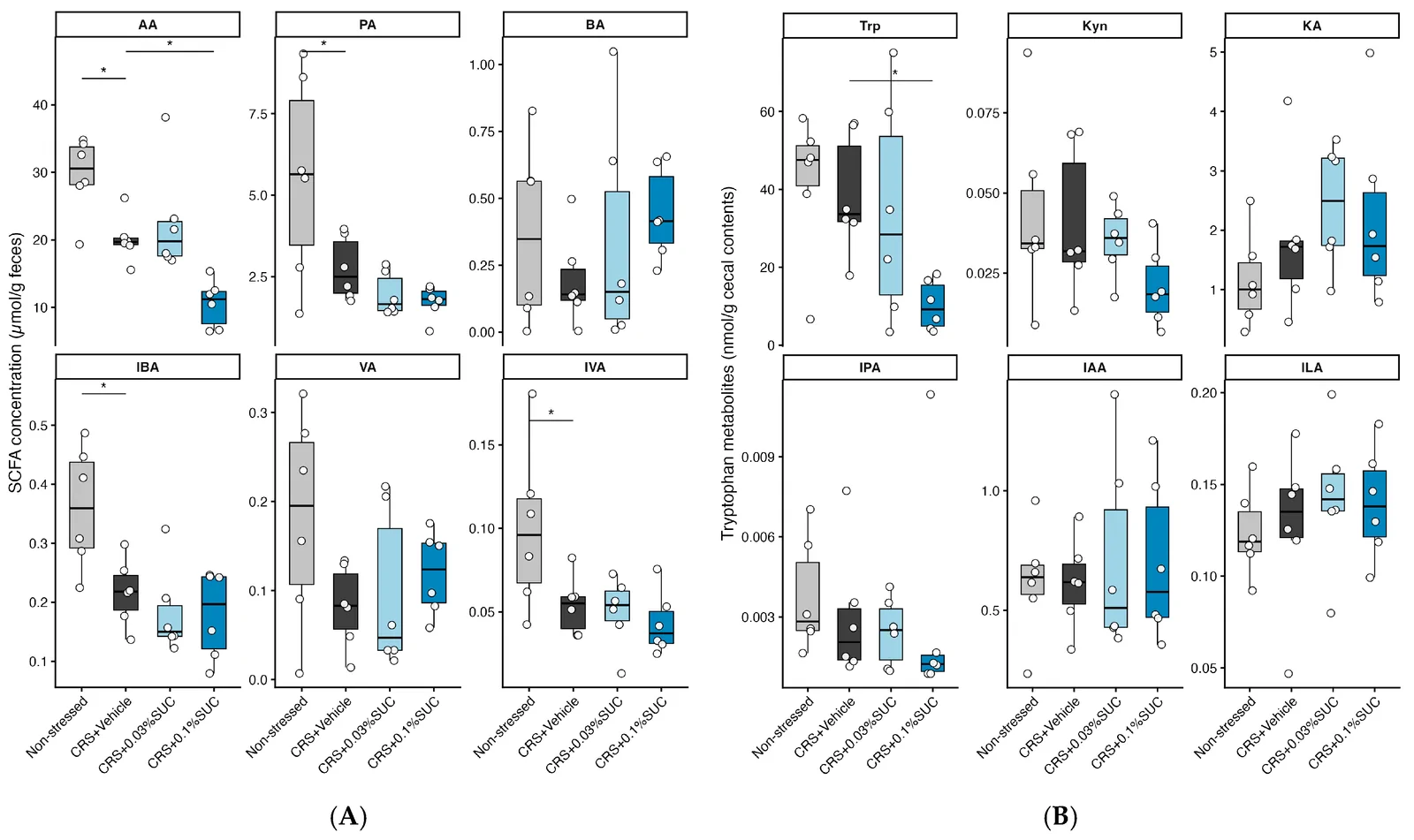
Sucralose exposure alters fecal SCFA profiles and cecal tryptophan-related metabolites in CRS-exposed mice. (**A**) Fecal concentrations of short-chain fatty acids (SCFAs) measured at the end of the experiment. The SCFAs analyzed included acetic acid (AA), propionic acid (PA), butyric acid (BA), isobutyric acid (IBA), valeric acid (VA), and isovaleric acid (IVA). (**B**) Cecal concentrations of tryptophan-related metabolites, including tryptophan (Trp), kynurenine (Kyn), kynurenic acid (KA), indole-3-propionic acid (IPA), indole-3-acetic acid (IAA), and indole-3-lactic acid (ILA). Mice were subjected to chronic restraint stress (CRS; 1 h/day) on Days 0–4 and 7–11. Immediately after the final restraint session on Day 11, mice received vehicle, 0.03% sucralose (SUC), or 0.1% SUC in drinking water until euthanasia on Day 23. Circles represent individual mice, and box plots indicate the median and interquartile range. Statistical comparisons were performed using one-way ANOVA followed by Dunnett’s multiple-comparison test with CRS + Vehicle as the reference group. Significance is indicated by *p* < 0.05 (*).

To determine whether sucralose exposure altered microbial metabolite profiles in CRS-exposed mice, we quantified fecal SCFAs and cecal tryptophan-related metabolites (Figure 4). Among the SCFAs analyzed, fecal AA concentrations were significantly lower in CRS + Vehicle mice than in Non-stressed mice and were further reduced in the CRS + 0.1% SUC group relative to the CRS + Vehicle group (Figure 4A). Fecal PA, IBA, and IVA levels were also significantly lower in CRS + Vehicle mice than in Non-stressed mice, whereas no significant differences were observed for BA or VA. Analysis of cecal tryptophan-related metabolites revealed that cecal Trp concentrations were significantly reduced in the CRS + 0.1% SUC group compared with CRS + Vehicle mice (Figure 4B). In contrast, concentrations of Kyn, KA, IPA, IAA, and ILA did not differ significantly among groups.

### 3.4. Sucralose Alters Colonic Epithelial Expression of Serotonergic and SCFA-Sensing Genes

**Figure 5:**
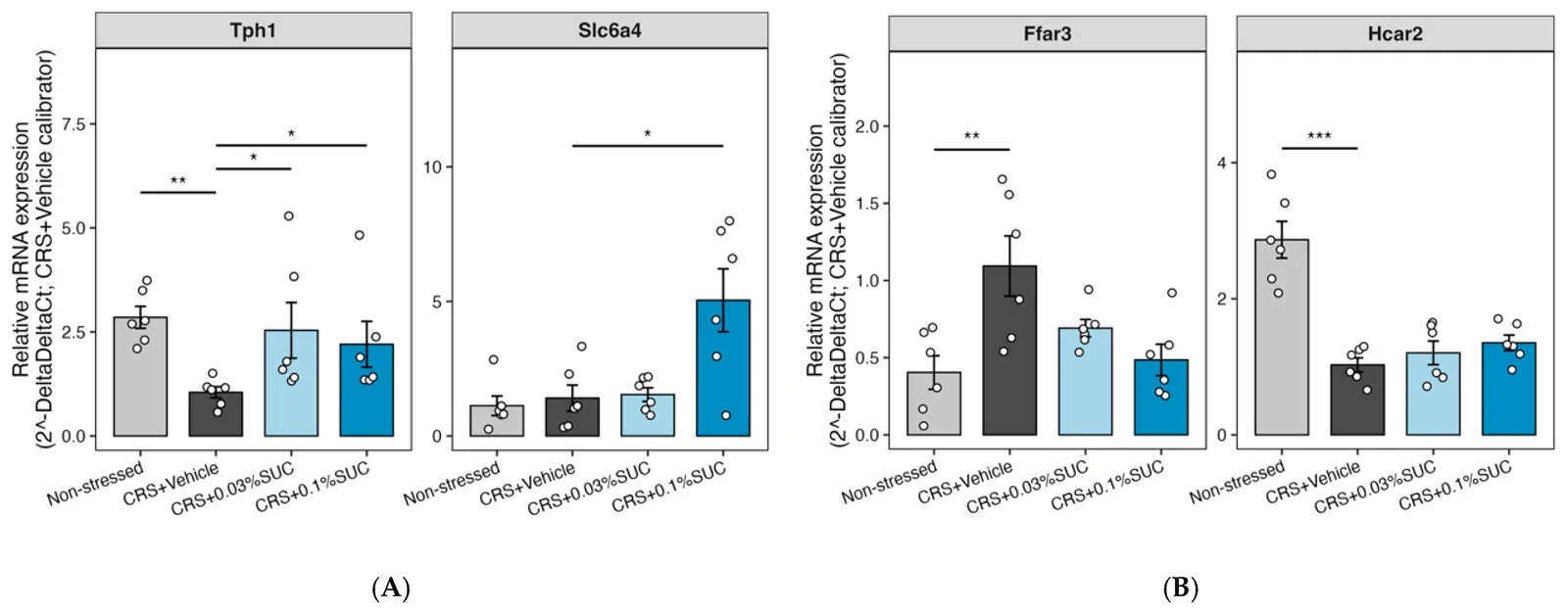
Sucralose alters colonic epithelial expression of genes associated with serotonin signaling and SCFA sensing in CRS-exposed mice. Relative mRNA expression of colonic epithelial genes associated with (**A**) serotonin signaling (*Tph1* and *Slc6a4*) and (**B**) short-chain fatty acid (SCFA) sensing (*Ffar3* and *Hcar2*). Mice were subjected to chronic restraint stress (CRS; 1 h/day) on Days 0–4 and 7–11. Immediately after the final restraint session on Day 11, mice received vehicle, 0.03% sucralose (SUC), or 0.1% SUC in drinking water until euthanasia on Day 23. Non-stressed mice received sterile drinking water without sucralose throughout the experimental period. Relative expression was calculated using the 2^−ΔΔCt^ method. Bars represent mean ± SEM, and circles represent individual mice. Statistical comparisons were performed on ΔCt values using one-way ANOVA followed by Dunnett’s multiple-comparison test, with CRS + Vehicle as the reference group. Significance is indicated by *p* < 0.05 (*), *p* < 0.01 (**), and *p* < 0.001 (***).

We next quantified colonic epithelial expression of genes associated with serotonin signaling and microbial metabolite sensing (Figure 5). Expression of the serotonin synthesis gene Tph1 was significantly reduced in CRS + Vehicle mice compared with Non-stressed controls and was significantly increased in both the CRS + 0.03% SUC and CRS + 0.1% SUC groups relative to CRS + Vehicle mice (Figure 5A). Expression of the serotonin transporter gene Slc6a4 was significantly increased in the CRS + 0.1% SUC group. Among SCFA-sensing receptors, Ffar3 expression was significantly elevated in CRS + Vehicle mice relative to Non-stressed controls, whereas Hcar2 expression was significantly decreased by CRS (Figure 5B).

### 3.5. Sucralose Modifies Behavioral and Serotonergic Responses to AITC-Induced Visceral Stimulation

**Figure 6:**
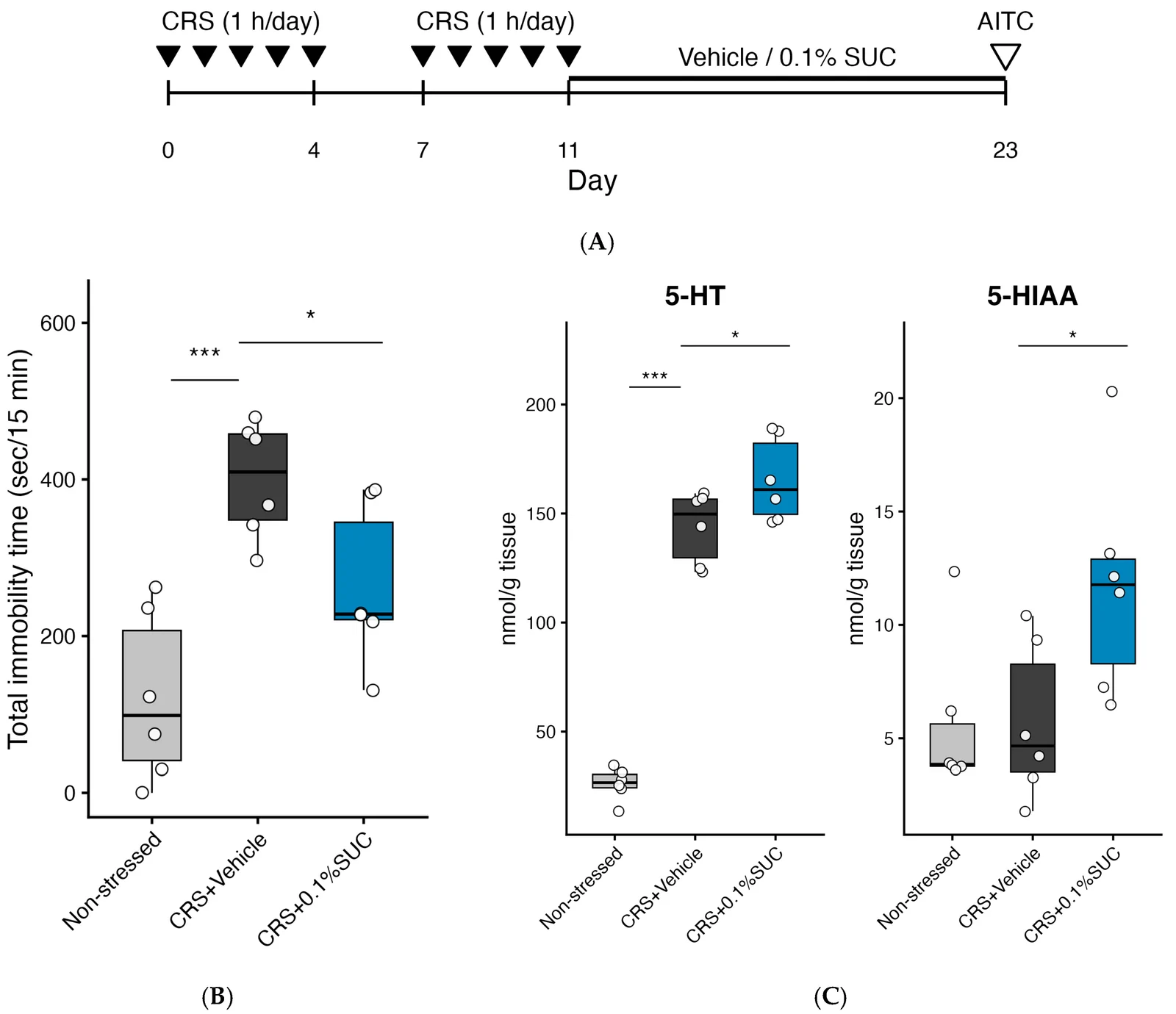
Treatment with 0.1% sucralose modifies behavioral and serotonergic responses to AITC-induced visceral stimulation in CRS-exposed mice. (**A**) Experimental design of the allyl isothiocyanate (AITC) challenge cohort. Mice were subjected to chronic restraint stress (CRS; 1 h/day) on Days 0–4 and 7–11. Black triangles indicate CRS sessions, and the open triangle indicates AITC administration. Immediately after the final restraint session on Day 11, mice received vehicle or 0.1% sucralose (SUC) in drinking water until euthanasia on Day 23. Non-stressed mice received sterile drinking water without sucralose throughout the experimental period. On Day 23, AITC was administered intracolonically, and mice were observed for 15 min before tissue collection. (**B**) Total immobility time during the 15 min observation period following AITC administration. Immobility behavior was quantified as a behavioral index of the response to AITC-induced visceral stimulation. (**C**) Concentrations of serotonin (5-HT) and its primary metabolite, 5-hydroxyindoleacetic acid (5-HIAA), in colonic epithelial scrapings collected immediately after the 15 min observation period. Box plots indicate the median and interquartile range, and circles represent individual mice. Statistical comparisons were performed using one-way ANOVA followed by Dunnett’s multiple-comparison test with CRS + Vehicle as the reference group. Significance is indicated by *p* < 0.05 (*) and *p* < 0.001 (***).

To evaluate behavioral and epithelial serotonergic responses following visceral stimulation, we performed an AITC challenge experiment using an independent cohort of mice (Figure 6A). Total immobility time following intracolonic AITC administration was significantly greater in CRS + Vehicle mice than in Non-stressed mice and was significantly lower in the CRS + 0.1% SUC group than in the CRS + Vehicle group (Figure 6B). We next quantified colonic epithelial 5-HT and its primary metabolite 5-HIAA in samples collected immediately after the 15 min behavioral observation period following AITC administration. Colonic epithelial 5-HT concentrations were increased in CRS + Vehicle mice compared with Non-stressed controls and were further elevated in the CRS + 0.1% SUC group (Figure 6C). Similarly, epithelial 5-HIAA concentrations were significantly increased in CRS + 0.1% SUC mice relative to CRS + Vehicle controls (Figure 6C).

Additional behavioral endpoints are shown in Supplementary Figure S1. Licking behavior exhibited a trend toward an increase in CRS-exposed mice compared with Non-stressed controls (*p* = 0.095), whereas 0.1% SUC treatment did not significantly modify this response. Crossing behavior was significantly reduced by CRS exposure but was not affected by SUC treatment. To determine whether SUC alone altered responses to visceral stimulation, an additional Non-stressed + 0.1% SUC cohort was examined (Supplementary Figure S2). In non-stressed mice, 0.1% SUC treatment did not significantly alter AITC-induced immobility, licking behavior, or crossing activity. Likewise, epithelial 5-HT and 5-HIAA concentrations following AITC stimulation were not significantly affected by 0.1% SUC treatment under non-stressed conditions (Supplementary Figure S2).

## 4. Discussion

In the present study, we investigated whether short-term SUC exposure modifies the intestinal ecosystem and host responses in mice previously exposed to chronic restraint stress. In CRS-exposed mice, SUC was associated with alterations in cecal microbial community structure, including reduced abundance of *Bacteroides acidifaciens*, lower fecal AA and cecal Trp concentrations, changes in colonic epithelial expression of serotonin- and SCFA-related genes, and altered behavioral and epithelial serotonergic responses following AITC-induced visceral stimulation. Together, these findings indicate that short-term experimental sucralose exposure was associated with microbial, metabolic, and epithelial alterations in mice previously exposed to chronic restraint stress.

The estimated SUC exposure was 39.4 ± 2.1 mg/kg/day in the 0.03% SUC group and 136.8 ± 8.4 mg/kg/day in the 0.1% SUC group. Both exposure levels, particularly that in the 0.1% SUC group, exceeded the acceptable daily intake established for humans. The present study was not designed to reproduce typical human dietary exposure, establish a human health risk threshold, or evaluate the safety of sucralose at customary intake levels. Rather, we used a short-term experimental exposure paradigm to determine whether sucralose could modify microbial, metabolic, and epithelial phenotypes after completion of the CRS protocol rather than during ongoing stress exposure. Accordingly, sucralose administration was initiated only after completion of the CRS protocol. Consequently, the present findings should not be interpreted as reflecting the effects of simultaneous stress and sucralose exposure. The concentrations were selected with reference to previous mouse studies reporting gut microbial alterations across a range of sucralose exposures [15,16,38]. Therefore, the present findings should be interpreted as evidence of biological responses under the specific experimental conditions used and should not be directly extrapolated to ordinary human sucralose consumption. Studies employing lower exposure levels, longer treatment periods, and designs that more closely reflect patterns of human intake will be required to establish the relevance of these findings to typical human sucralose consumption. Future studies directly comparing concurrent and post-stress sucralose exposure will also be required to determine how the timing of dietary exposure influences host–microbiota interactions.

Most of the observed alterations were more apparent in the 0.1% SUC group, suggesting that several intestinal responses were more pronounced at the higher experimental exposure level. In an additional non-stressed cohort, 0.1% SUC did not significantly alter AITC-induced behavioral responses or post-stimulation epithelial 5-HT and 5-HIAA concentrations. However, because microbiota composition, intestinal metabolite profiles, and epithelial gene expression were not evaluated in non-stressed mice receiving sucralose, the present study cannot determine whether these changes required prior CRS exposure or represented direct effects of sucralose. Previous studies have reported that sucralose alone can alter gut microbial composition in non-stressed mice [15,16,38], indicating that microbial effects are not necessarily specific to stress-associated conditions and highlighting the importance of directly comparing stressed and non-stressed animals in future investigations. In contrast, the altered behavioral and post-stimulation epithelial serotonergic responses were observed in the CRS AITC cohort but were not detected in the separately examined non-stressed mice receiving 0.1% sucralose, suggesting that the stress-associated physiological context may have contributed to these particular host responses.

A prominent finding of the present study was the association between sucralose exposure and cecal microbial community composition. The overall PERMANOVA indicated significant differences among the experimental groups; however, the pairwise evidence differed by dose. The CRS + 0.03% sucralose group differed significantly from the CRS + Vehicle group after multiple-testing correction, whereas the comparison involving the CRS + 0.1% sucralose group narrowly missed the adjusted significance threshold. Therefore, the higher-dose group should not be interpreted as showing a statistically significant community-wide shift. Rather, the microbiome effect at 0.1% sucralose was more clearly supported by taxon-specific analyses, particularly the reduction in *Bacteroides acidifaciens*. Although alpha diversity was largely preserved, these findings indicate that sucralose exposure was associated with changes in microbial community composition without marked alterations in overall within-sample diversity. Species-level analysis identified *B. acidifaciens* as the taxon showing the strongest association with SUC exposure. Both MaAsLin2 and species-level abundance analyses supported a reduction in *B. acidifaciens*, with the strongest evidence observed in the 0.1% SUC group. Previous studies have suggested that this species contributes to host metabolic regulation and intestinal homeostasis [39,40], although its role in stress-associated intestinal alterations remains poorly understood. The consistent reduction in *B. acidifaciens* observed in the present study therefore represents a characteristic microbial alteration associated with short-term sucralose exposure in CRS-exposed mice. However, the present data do not establish that this organism directly mediates the observed alterations in microbial metabolites, epithelial serotonergic responses, or behavioral outcomes. Accordingly, the reduction in *B. acidifaciens* should be interpreted as an association rather than evidence of a causal mechanism. Future studies incorporating microbiota-transfer approaches, *B. acidifaciens* supplementation, or other mechanistic interventions will be required to determine whether this organism contributes directly to the observed microbial, epithelial serotonergic, and behavioral alterations in the present model. In contrast, associations involving *Parabacteroides goldsteinii* and *Parabacteroides distasonis* were observed only at an exploratory FDR threshold and should be interpreted cautiously.

One of the notable metabolic changes observed in the present study was the reduction in fecal acetate and cecal tryptophan concentrations following SUC exposure. Acetate is the most abundant short-chain fatty acid in the intestine and plays important roles in epithelial homeostasis, microbial cross-feeding, and host–microbiota communication [6]. Tryptophan is similarly important because it serves as a precursor for serotonin synthesis and a substrate for multiple host- and microbiota-dependent metabolic pathways [5]. The mechanisms underlying the reductions in acetate and tryptophan remain unclear. However, because these metabolic changes occurred in parallel with alterations in microbial community structure, they are consistent with remodeling of the intestinal metabolic environment following SUC exposure. Importantly, the present findings do not establish direct relationships between specific bacterial taxa and the observed changes in acetate or tryptophan concentrations. Instead, these metabolites are more likely to reflect the collective metabolic activity of the intestinal microbial community and its interactions with the host.

Notably, the observed microbial and metabolic alterations occurred in the absence of detectable changes in intestinal permeability. FITC-dextran permeability was unchanged. Although CRS significantly increased epithelial *Lcn2* expression, sucralose did not further alter the expression of *Il6*, *Lcn2*, or *Ptgs2* beyond that induced by CRS. In addition, several epithelial genes associated with barrier function and mucosal homeostasis exhibited selective rather than widespread changes. Together, these findings indicate that SUC did not produce widespread additional changes in epithelial barrier or inflammation-related markers beyond those associated with CRS.

Despite the absence of additional inflammation-related gene-expression changes, significant colon shortening was observed in the 0.1% SUC group. Colon shortening is widely used as a gross morphological indicator of intestinal pathology in experimental models; however, in the present study it occurred in the absence of measurable barrier dysfunction or widespread inflammation-related gene-expression changes. Therefore, the reduced colon length should be interpreted as an isolated anatomical finding rather than evidence of overt inflammatory injury, and its biological significance remains uncertain. Although the mechanism underlying this observation remains unresolved, the absence of marked barrier disruption is consistent with the concept that altered host responsiveness to luminal stimuli can occur without overt epithelial injury, a feature that has been described in IBS [2,41].

A major finding of the present study was that SUC modified epithelial serotonergic responses following AITC-induced visceral stimulation. CRS reduced expression of the serotonin synthesis gene *Tph1*, whereas SUC treatment partially restored Tph1 expression and increased expression of the serotonin transporter gene *Slc6a4*, particularly in the 0.1% SUC group. More importantly, epithelial concentrations of both 5-HT and its primary metabolite 5-HIAA were elevated following AITC stimulation in sucralose-treated CRS mice. Given the established role of serotonin in gut–brain communication and visceral sensory signaling [2,7,8], these findings are consistent with the possibility that SUC was associated with altered host responses to luminal stimulation. Because these measurements were obtained after AITC administration, the findings are most consistent with altered stimulus-evoked serotonergic responses rather than changes in basal serotonin homeostasis. The concurrent increase in 5-HIAA is compatible with increased serotonin turnover following stimulation. Notably, these effects were not observed in non-stressed mice receiving SUC alone, supporting the possibility that prior stress exposure contributed to the serotonergic phenotype associated with sucralose treatment.

In the same cohort, AITC-induced immobility behavior was reduced in SUC-treated mice. Interpretation of this finding requires caution because immobility in the present paradigm likely reflects a composite response involving nociceptive processing, stress-related behavior, and locomotor activity rather than a direct measure of visceral pain intensity. Therefore, the reduced immobility observed in SUC-treated mice should not necessarily be interpreted as an improvement in visceral hypersensitivity. Moreover, because crossing activity was not significantly altered by sucralose treatment, the reduced immobility cannot be explained solely by increased locomotor activity. Instead, the combined behavioral and biochemical findings suggest that sucralose was associated with altered host responsiveness to visceral stimulation, although the precise physiological significance of this altered response remains to be determined. Additional assessment of locomotor activity, anxiety-related behavior, and colorectal distension-based visceral nociception will be required to clarify the mechanisms underlying the altered behavioral phenotype. Future studies incorporating pharmacological modulation of serotonergic signaling or receptor-specific pharmacological inhibition will also be required to determine whether epithelial serotonin directly contributes to the behavioral phenotype observed following AITC stimulation.

A seemingly paradoxical observation was that enhanced epithelial serotonergic responses occurred despite reduced cecal Trp concentrations. This finding suggests that luminal tryptophan availability and stimulus-evoked epithelial serotonergic responses are not necessarily directly coupled. Because microbiota composition, microbial metabolites, and serotonergic responses were not evaluated within the same animals, the relationships among these observations could not be assessed at the individual-animal level. Future studies integrating matched microbiome, metabolomic, and functional datasets will be required to determine whether the observed microbiota alterations contribute causally to altered serotonergic responsiveness following SUC exposure.

Several limitations should be acknowledged. First, this study was designed to evaluate the effects of short-term SUC exposure using experimental exposure levels that exceeded the ADI values established by regulatory agencies. Therefore, the present findings do not directly address the consequences of long-term consumption or exposure at typical human intake levels. Second, microbiota analyses were based on 16S rRNA gene sequencing and therefore provide limited insight into the functional pathways responsible for the observed alterations in microbial metabolites and host responses. Third, although *Bacteroides acidifaciens* was consistently reduced following sucralose exposure, causal relationships between specific bacterial taxa, microbial metabolites, and epithelial serotonergic responses were not established. Future studies incorporating metagenomic, metabolomic, and microbiota-transfer approaches will be required to determine whether the observed microbiota alterations contribute causally to the physiological alterations observed in this model. Fourth, the behavioral and serotonergic analyses were performed in an independent AITC challenge cohort in which microbiota composition and intestinal metabolite profiles were not evaluated. Consequently, it remains unclear whether the altered serotonergic responses observed following AITC stimulation were directly linked to the microbial and metabolic changes identified in the primary cohort. Fifth, serotonergic measurements were obtained only after AITC stimulation and therefore do not distinguish whether SUC altered basal serotonin homeostasis or specifically modified stimulus-evoked serotonergic responses. Sixth, although the sample size used in this study was comparable to that of previous exploratory mouse studies, no formal a priori power analysis was performed. Therefore, the relatively small sample size may have limited statistical power, particularly for negative findings and modest effect sizes, and larger independent studies will be required to confirm the robustness of the present observations. Seventh, microbiome, metabolomic, and epithelial gene-expression datasets were obtained from the same animals in the primary cohort, whereas behavioral and post-stimulation serotonergic measurements were obtained from an independent AITC cohort. Consequently, fully integrated microbiome–metabolome–gene-expression–behavior network analyses could not be performed at the individual-animal level. Future studies using matched multi-omics and functional datasets from the same animals will enable more comprehensive mechanistic analyses. Eighth, inflammatory responses were evaluated at the mRNA level only. Therefore, the present study cannot determine whether the observed transcriptional changes were accompanied by corresponding changes in protein expression or biological activity. Future studies incorporating protein-level analyses, such as immunoblotting or immunohistochemistry, will be important to further characterize the inflammatory responses associated with sucralose exposure.

## 5. Conclusions

In conclusion, short-term sucralose exposure in mice previously exposed to CRS was associated with colon shortening, alterations in cecal microbial community structure, reduced abundance of *Bacteroides acidifaciens*, decreased fecal acetate and cecal tryptophan concentrations, and altered epithelial serotonergic responsiveness following visceral stimulation. The behavioral and post-stimulation serotonergic alterations were not observed in non-stressed mice exposed to SUC alone, suggesting that prior stress exposure may influence these host responses to sucralose. However, because microbial and metabolic effects were not evaluated in non-stressed mice receiving SUC, their dependence on the stress context remains unclear. Together, our findings suggest that short-term sucralose exposure is associated with microbial, metabolic, and epithelial alterations following chronic stress exposure and identify epithelial serotonergic responsiveness as a candidate host phenotype warranting further mechanistic investigation. Although direct extrapolation to humans is not possible, the present findings provide a preclinical basis for future translational studies examining how dietary sweeteners influence host–microbiota interactions following chronic stress exposure.

## Data Availability

Raw 16S rRNA gene sequencing data have been deposited in the NCBI Sequence Read Archive under BioProject accession number PRJNA1477735 and will be publicly released upon publication.

## Supplementary Materials

The following supporting information can be downloaded at: https://www.mdpi.com/article/10.3390/nu18162660/s1, Figure S1: Additional behavioral responses following AITC-induced visceral stimulation in non-stressed and CRS-exposed mice treated with vehicle or 0.1% sucralose; Figure S2: No significant differences in behavioral or epithelial serotonergic responses to AITC-induced visceral stimulation were observed between non-stressed vehicle- and 0.1% sucralose-treated mice; Table S1: Primer sequences used for quantitative real-time PCR; Table S2: Optimized MRM transitions and compound-specific parameters for 3-NPH–derivatized SCFAs; Table S3: Optimized MRM transitions and compound-specific parameters for tryptophan-related metabolites; Table S4: Descriptive statistics, effect estimates, and standardized effect sizes for the principal outcomes of the study.
