# Supplementary material for: Sucralose Alters Gut Microbiota, Intestinal Metabolites, and Epithelial Serotonergic Responses to Visceral Stimulation in Mice Exposed to Chronic Restraint Stress
Source: Nutrients. 2026 Aug 14;18(16):2660. doi: 10.3390/nu18162660 (PMC13516141; doi:10.3390/nu18162660)
Supplement: Supplementary file 1 [file nutrients-18-02660-s001.zip › nutrients-4491589-supplementary.pdf]

Supplementary Materials

This file contains Supplementary Figures S1−S2 and Supplementary Tables S1–S4 supporting the findings of the present study.

A.

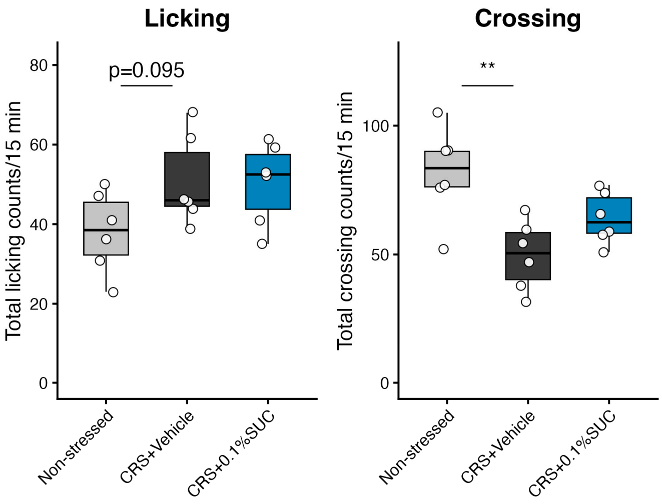

Figure S1. Additional behavioral responses following AITC-induced visceral stimulation in non-stressed and CRS-exposed mice treated with vehicle or 0.1% sucralose.

Behavioral responses during the 15-min observation period following intracolonic administration of allyl isothiocyanate (AITC). Total licking counts and total crossing counts were quantified as additional indices of the behavioral response to visceral stimulation. Mice were subjected to chronic restraint stress (CRS; 1 h/day) on Days 0–4 and 7–11. Immediately after completion of the CRS protocol—that is, after the final restraint session on Day 11—mice received vehicle or 0.1% sucralose (SUC) in drinking water until euthanasia on Day 23. Non-stressed mice received sterile drinking water without sucralose throughout the experimental period. Box plots indicate the median and interquartile range, and individual points represent individual mice. Statistical comparisons were performed using one-way ANOVA followed by Dunnett's multiple-comparison test with CRS + Vehicle as the reference group. Significance is indicated by $p < 0.01$ (**).

A.

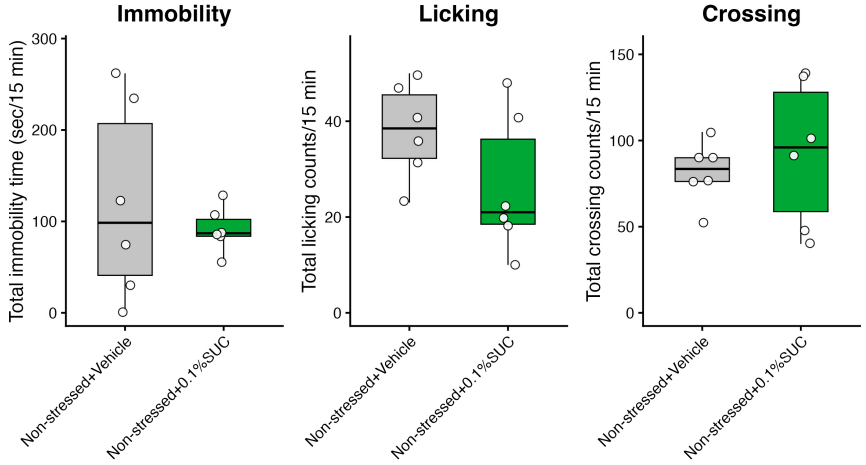

B.

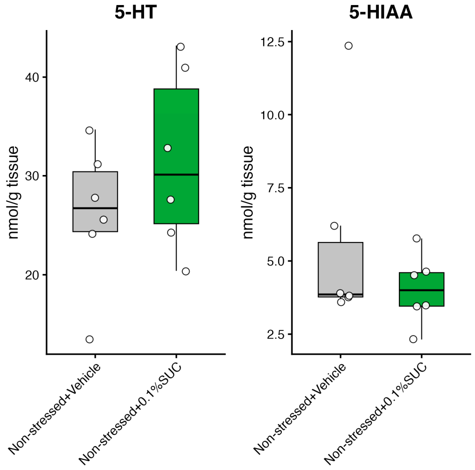

Figure S2. No significant differences in behavioral or epithelial serotonergic responses to AITC-induced visceral stimulation were observed between non-stressed vehicle- and 0.1% sucralose-treated mice.

(A) Allyl isothiocyanate (AITC)-induced behavioral responses in Non-stressed + Vehicle and Non-stressed + 0.1% sucralose (SUC) mice. Total immobility time, licking counts, and crossing counts were quantified during the 15-min observation period following intracolonic administration of AITC. (B) Concentrations of serotonin (5-HT) and its primary metabolite, 5-hydroxyindoleacetic acid (5-HIAA), in colonic epithelial scrapings collected immediately after the 15-min observation period. Box plots indicate the median and interquartile range, and individual points represent individual mice. Statistical comparisons were performed using Welch's two-tailed unpaired t-test. No statistically significant differences were detected between the two groups. The

Non-stressed + Vehicle group corresponds to the same Non-stressed group presented in Figure 6 and is included here for comparison with the additional Non-stressed + 0.1% SUC group.

Supplementary tables

Table S1. Primer sequences used for quantitative real-time PCR.

Gene	Primer	Sequence (5′–3′)
Gapdh	Forward	AGAAGGTGGTGAAGCAGGCATC
Gapdh	Reverse	CGAAGGTGGAAGAGTGGGAGTTG
Tph1	Forward	CAGTGGCTCTGAGGTGAGTG
Tph1	Reverse	AGTTGATCCCGTCCTTGCTG
Slc6a4	Forward	CAAAACGTCTGGCAAGGTGG
Slc6a4	Reverse	ACACCCCTGTCTCCAAGAGT
Ffar3	Forward	TATGTCAGCCGTGAGAGTCCGT
Ffar3	Reverse	CCAGGAGTTGATGAAAGTCGGC
Hcar2	Forward	CTGTTTCCACCTCAAGTCCTGG
Hcar2	Reverse	CATAGTTGTCCGTCAGGAACGG
Ocln	Forward	CTATGGGACAGGGCTCTTTGGA
Ocln	Reverse	AGGAAGCGATGAAGCAGAAGGC
Tjp1	Forward	AATGAGGATGAGGTTGTGTC
Tjp1	Reverse	TTGTAGTTGTGAAGAGATGGTG
Klf4	Forward	GTGCCCCGACTAACCGTTG
Klf4	Reverse	GTCGTTGAACTCCTCGGTCT
Tff3	Forward	TCCAAGCCAATGTATGGTGCCG
Tff3	Reverse	CAGGGCACATTTGGGATACTGG
Muc2	Forward	ATGCCCACCTCCTCAAAGAC
Muc2	Reverse	GTAGTTTCCGTTGGAACAGTGAA
Il6	Forward	TCACAGAAGGAGTGGCTAAGGACC
Il6	Reverse	ACGCACTAGGTTTGCCGAGTAGAT
Lcn2	Forward	ATGTCACCTCCATCCTGGTCAG

Lcn2	Reverse	GCCACTTGCACATTGTAGCTCTG
Ptgs2	Forward	GCGAGCTAAGAGCTTCAGGA
Ptgs2	Reverse	TCATACATTCCCCACGGTTT

Table S2. Optimized MRM transitions and compound-specific parameters for 3-NPH–derivatized SCFAs.

Compound	Type	Q1 (m/z)	Q3 (m/z)	DP (V)	CE (V)	RT (min)
AA-3NPH	Quantifier	193.9	150.8	−55	−18	4.2
PA-3NPH	Quantifier	207.9	164.9	−55	−18	7.4
BA-3NPH	Quantifier	222.0	179.0	−80	−20	9.2
IBA-3NPH	Quantifier	222.0	179.0	−80	−20	9.0
VA-3NPH	Quantifier	236.0	193.0	−90	−20	10.7
IVA-3NPH	Quantifier	236.0	193.0	−90	−20	10.4
AA-$^{13}C_2$-3NPH	Internal standard	195.9	136.9	−55	−24	4.2
PA-$^{13}C_3$-3NPH	Internal standard	210.9	152.0	−55	−20	7.4
BA-$^{13}C_4$-3NPH	Internal standard	226.0	136.7	−80	−26	9.2

Multiple reaction monitoring (MRM) quantifier transitions, declustering potentials (DP), collision energies (CE), and retention times for 3-nitrophenylhydrazine (3-NPH)-derivatized short-chain fatty acids (SCFAs) and isotope-labeled internal standards used for LC–MS/MS quantification in negative-ion mode. RT, retention time; AA, acetic acid; PA, propionic acid; BA, butyric acid; IBA, isobutyric acid; VA, valeric acid; IVA, isovaleric acid.

Table S3. Optimized MRM transitions and compound-specific parameters for tryptophan-related metabolites.

Compound	Type	Q1 (m/z)	Q3 (m/z)	DP (V)	CE (V)	RT (min)
Kyn	Quantifier	209.1	94.1	91	19	1.4

Trp	Quantifier	205.1	91.1	41	55	2.9
KA	Quantifier	190.0	89.0	106	55	6.2
ILA	Quantifier	206.0	130.0	56	47	6.7
IAA	Quantifier	176.1	103.0	51	43	7.2
IPA	Quantifier	190.1	77.2	51	67	8.4
Kyn-d4	Internal standard	213.1	96.1	31	17	1.4
Trp-d5	Internal standard	210.2	122.1	21	35	2.9
IAA-d5	Internal standard	181.1	134.0	46	23	7.2

Multiple reaction monitoring (MRM) quantifier transitions, declustering potentials (DP), collision energies (CE), and retention times for tryptophan-related metabolites and isotope-labeled internal standards used for LC–MS/MS quantification in positive-ion mode. RT, retention time; Trp, tryptophan; Kyn, kynurenine; KA, kynurenic acid; IAA, indole-3-acetic acid; ILA, indole-3-lactic acid; IPA, indole-3-propionic acid.

Table S4. Descriptive statistics, effect estimates, and standardized effect sizes for the principal outcomes of the study.

Figure	Outcome	Comparison	Comparison group, mean ± SD	Reference group, mean ± SD	Effect estimate (95% CI)	Cohen's *d*	Dunnett-adjusted *p* value
Figure 1B	Colon length	Non-stressed vs CRS+Vehicle	60.17 ± 2.86	62.17 ± 3.31	-2.00 (-6.77 to 2.77)	-0.65	0.578
Figure 1B	Colon length	CRS+0.03%SUC vs CRS+Vehicle	61.00 ± 4.34	62.17 ± 3.31	-1.17 (-5.93 to 3.60)	-0.30	0.839
Figure 1B	Colon length	CRS+0.1%SUC vs CRS+Vehicle	57.17 ± 1.94	62.17 ± 3.31	-5.00 (-9.77 to -0.23)	-1.84	0.038
Figure 3D	*Bacteroides acidifaciens* relative abundance	Non-stressed vs CRS+Vehicle	8.06 ± 5.14	8.79 ± 3.28	0.83 (0.38 to 1.79)	-0.37	0.838
Figure 3D	*Bacteroides acidifaciens* relative abundance	CRS+0.03%SUC vs CRS+Vehicle	4.85 ± 2.55	8.79 ± 3.28	0.56 (0.26 to 1.22)	-1.49	0.174
Figure 3D	*Bacteroides acidifaciens* relative abundance	CRS+0.1%SUC vs CRS+Vehicle	4.04 ± 3.06	8.79 ± 3.28	0.44 (0.20 to 0.95)	-1.63	0.034
Figure 4A	Fecal acetate	Non-stressed vs CRS+Vehicle	29.59 ± 5.75	20.14 ± 3.43	9.44 (1.32 to 17.57)	1.99	0.021
Figure 4A	Fecal acetate	CRS+0.03%SUC vs CRS+Vehicle	22.56 ± 8.01	20.14 ± 3.43	2.41 (-5.71 to 10.54)	0.39	0.767
Figure 4A	Fecal acetate	CRS+0.1%SUC vs CRS+Vehicle	10.57 ± 3.49	20.14 ± 3.43	-9.57 (-17.70 to -1.44)	-2.76	0.019
Figure 4B	Cecal tryptophan	Non-stressed vs CRS+Vehicle	41.88 ± 18.37	38.36 ± 15.41	3.52 (-24.28 to 31.32)	0.21	0.958
Figure 4B	Cecal tryptophan	CRS+0.03%SUC vs CRS+Vehicle	34.19 ± 28.35	38.36 ± 15.41	-4.17 (-31.97 to 23.63)	-0.18	0.940

Figure 4B	Cecal tryptophan	CRS+0.1%SUC vs CRS+Vehicle	10.20 ± 6.33	38.36 ± 15.41	-28.16 (-55.96 to -0.36)	-2.39	0.047
Figure 5A	Colonic epithelial *Tph1* mRNA expression	Non-stressed vs CRS+Vehicle	2.85 ± 0.65	1.05 ± 0.33	2.79 (1.47 to 5.30)	-3.49	0.002
Figure 5A	Colonic epithelial *Tph1* mRNA expression	CRS+0.03%SUC vs CRS+Vehicle	2.54 ± 1.64	1.05 ± 0.33	2.18 (1.15 to 4.14)	-1.63	0.015
Figure 5A	Colonic epithelial *Tph1* mRNA expression	CRS+0.1%SUC vs CRS+Vehicle	2.20 ± 1.35	1.05 ± 0.33	1.96 (1.03 to 3.72)	-1.56	0.039
Figure 5A	Colonic epithelial *Slc6a4* mRNA expression	Non-stressed vs CRS+Vehicle	1.12 ± 0.89	1.41 ± 1.18	0.88 (0.27 to 2.83)	0.14	0.970
Figure 5A	Colonic epithelial *Slc6a4* mRNA expression	CRS+0.03%SUC vs CRS+Vehicle	1.54 ± 0.62	1.41 ± 1.18	1.42 (0.44 to 4.57)	-0.48	0.760
Figure 5A	Colonic epithelial *Slc6a4* mRNA expression	CRS+0.1%SUC vs CRS+Vehicle	5.04 ± 2.86	1.41 ± 1.18	3.97 (1.24 to 12.75)	-1.50	0.018
Figure 6B	Total immobility time	Non-stressed vs CRS+Vehicle	120.67 ± 107.71	399.50 ± 74.30	-278.83 (-414.60 to -143.06)	-3.01	<0.001
Figure 6B	Total immobility time	CRS+0.1%SUC vs CRS+Vehicle	262.83 ± 101.81	399.50 ± 74.30	-136.67 (-272.44 to -0.90)	-1.53	0.048
Figure 6C	Colonic epithelial 5-HT	Non-stressed vs CRS+Vehicle	26.13 ± 7.32	143.97 ± 16.28	-117.84 (-138.97 to -96.72)	-9.34	<0.001
Figure 6C	Colonic epithelial 5-HT	CRS+0.1%SUC vs CRS+Vehicle	165.88 ± 18.62	143.97 ± 16.28	21.91 (0.79 to 43.03)	1.25	0.042
Figure 6C	Colonic epithelial 5-HIAA	Non-stressed vs CRS+Vehicle	5.61 ± 3.45	5.69 ± 3.43	-0.08 (-5.77 to 5.61)	-0.02	0.998
Figure 6C	Colonic epithelial 5-HIAA	CRS+0.1%SUC vs CRS+Vehicle	11.78 ± 4.96	5.69 ± 3.43	6.09 (0.40 to 11.79)	1.43	0.036

Values are presented as mean ± SD. All comparisons were performed against the CRS + Vehicle group using Dunnett's multiple-comparison test. Effect estimates are presented as mean differences for continuous outcomes, fold changes for qPCR outcomes ($2^{-\Delta\Delta Ct}$), and abundance ratios for *Bacteroides acidifaciens* relative abundance. Cohen's *d* values for qPCR outcomes were calculated using ΔCt values; therefore, a negative Cohen's *d* indicates higher relative expression in the comparison group because lower ΔCt values correspond to higher expression. Cohen's *d* values for *B. acidifaciens* were calculated using log10-transformed relative abundance values. Each experimental group consisted of six mice.
